# Advances in brain barriers and brain fluid research and news from* Fluids and Barriers of the CNS*

**DOI:** 10.1186/s12987-016-0026-1

**Published:** 2016-01-28

**Authors:** Lester R. Drewes, Hazel C. Jones, Richard F. Keep

**Affiliations:** 1 Department of Biomedical Sciences, University of Minnesota Medical School, Duluth, MN 55812 USA; 2 Gagle Brook House, Chesterton, Bicester, OX26 1UF UK; 3 Department of Neurosurgery, University of Michigan, Ann Arbor, MI 48105 USA

## Abstract

Research into brain barriers and brain fluids has been advancing rapidly in recent years. This editorial aims to highlight some of the advances that have improved our understanding of this complex subject. It also brings you news of developments for Fluids and Barriers of the CNS including a new affiliation between the journal and the International Society for Hydrocephalus and CSF disorders.

## The field of brain fluids and brain barriers

During the past 24 months there has continued to be an up-swell in interest in neurovascular unit (NVU)/blood–brain barrier (BBB) and cerebrospinal fluid (CSF) research. That interest has led to many new and important findings. For example, there continues to be an increasing interest in pericytes at the NVU. New experiments [1] support initial findings [2, 3] on the importance of pericytes in BBB development, including regulation of endothelial transcytosis. There is also evidence that pericytes may have a role in control of cerebral blood flow [4] and are a target in ischemic stroke [4, 5]. Pericytes are now also being integrated into in vitro BBB models with some co-cultures showing permeabilities close to those in vivo [6]. The use of human-derived pluripotent stem cells to produce endothelial cells, astrocytes and pericytes to develop in vitro NVU/BBB models holds great promise for the future [6, 7].

Fluid flow within the brain parenchyma continues to be an area of intense research. The glymphatic hypothesis, with perivascular flow into and out of the brain and astrocytic aquaporin-4 linked flow within the brain [8–11], continues to generate important data on the clearance of compounds from the brain in disease (e.g. [12, 13]) as well as the effects of parameters such as sleep and posture on such flow [14, 15]. There is also growing evidence that the brain has lymphatic vessels within the dura mater and these may be important in brain fluid drainage [16]. It should be noted, however, that there continues to be data showing that, in some regions, brain interstitial fluid flows to the cerebral ventricles and that (in rodents) a major outflow of CSF is via the subarachnoid space and across the cribriform plate [17]. In addition, while interstitial fluid flow may be important in clearing compounds such as amyloid from the brain, the importance of clearance at the NVU/BBB should not be overlooked and may be more important [18]. Advances in imaging techniques have contributed greatly to our understanding of the brain barriers and interstitial and CSF flow. Two-photon imaging has contributed greatly to animal studies (e.g. [9, 19] and novel magnetic resonance imaging (MRI) has led to evidence that inspiration has a greater effect on CSF flow than arterial pulsation [20]. This research is of great importance for hydrocephalus and other CSF disorders. Hydrocephalus in various forms can arise at different stages of life (perinatal, mid-life and in the elderly), all with different and complex pathologies, none of which are fully understood. Surgical treatment for hydrocephalus has been practiced, with many variations, for about 50 years and has been beset with complications. Basic research on fluid flow into, within and out of the brain and on the removal of waste products is extremely important for improving treatment for patients.

The field of drug delivery across the BBB and blood–CSF barrier continues to present major challenges. There is growing interest in using focused ultrasound to transiently disrupt the NVU/BBB for drug delivery [21] and there is increasing evidence for the use intranasal delivery to circumvent the NVU/BBB [22]. As in any field, there are areas of controversy. For example, these include the relative role of the choroid plexus in brain fluid production and the reader is referred to a number of reviews in this and other journals [10, 11, 23]. There is also renewed debate about the relative roles of different pathways in NVU/BBB disruption in disease. For example, in stroke, there is discussion about the relative role of tight junction disruption, transcytosis and endothelial cell death in the increased barrier permeability that occurs after cerebral ischemia [19, 24, 25]. Such debates are important in any field and are a spur for the development of new experimental approaches and technologies. There are also findings that cause marked surprise and promote new avenues for research. Thus, Braniste et al. found that germ-free mice, beginning in utero, have increased barrier permeability compared to pathogen-free mice with a normal gut microbiome [26]. These results suggest novel links between the gut microbiome and brain, a growing field of research.

One ultimate goal of biomedical research is the development of new therapies. An area where new treatments are desperately needed is malignant cerebral edema, which is often associated with NVU/BBB disruption. It is, therefore, important to note that glyburide (glibenclamide) is now in phase II clinical trial for such edema in stroke (NCT01794182). Glyburide targets the sulfonylurea-1-transient receptor potential melastatin-4 (Sur1-Trpm4) channel that is upregulated after stroke, including in cerebral endothelial cells [27, 28].

## Journal metrics

All of these exciting areas of research are within the scope of* Fluids and Barriers of the CNS* (FBCNS). During 2015, FBCNS published on a wide range of topics covering different aspects of function and pathology of the NVU, the CSF response to pathogens, cancer metastasis in the brain, MRI imaging in hydrocephalus, and brain interstitial fluid flow. The average number of journal accesses per month in 2015 taken from the BioMed Central website was* 43,132*. The most highly accessed papers in 2015 were: 12:3:* The effects of Beta-Endorphin: state change modification* [29]; 12:16:* A new glaucoma hypothesis: a role of glymphatic system dysfunction* [30]; 12:4:* Differential cytokine expression by brain microglia/macrophages in primary culture after oxygen glucose deprivation and their protective effects on astrocytes during anoxia* [31]; and 12:5:* Vascular endothelial growth factor blockade alters magnetic resonance imaging biomarkers of vascular function and decreases barrier permeability in a rat model of lung cancer brain metastasis* [32].

In the past 3 years the number of citations for articles in FBCNS has risen steadily, as has the SCImago journal rank. The most highly cited article is* Johanson et al: Multiplicity of cerebrospinal fluid functions: New challenges in health and disease* [33].

## Journal affiliations

The Editors of *Fluids and Barriers of the CNS* are very pleased to announce that FBCNS is now affiliated to the International Society for Hydrocephalus and CSF Disorders (ISHCSF). This is in addition to affiliation to the International Brain Barriers Society (IBBS) set up in 2014. Thus *Fluids and Barriers of the CNS* is an official journal for both societies, this being a reflection of the full scope of the journal content. Society members enjoy a substantial discount on the article submission fee needed to cover open access publishing costs. The ISHCSF was inaugurated in September 2008 and is registered as a non-profit organization http://ishcsf.com. From the beginning, its mission has been to advance the art and science of the field of clinical care and research into hydrocephalus and CSF disorders, and thereby promote the best possible care for patients. It holds annual meetings and its aims are to promote international exchange, encourage worldwide representation and to stimulate research and debate. The Society supports standardized methods and ethically-conducted clinical research and basic research in hydrocephalus, CSF disorders and related fields. In previous years, FBCNS has been interacting with ISHCSF through publication of a collection of members’ papers: thematic series* Hydrocephalus: Promoting Research for Improved Outcomes* (http://www.biomedcentral.com/collections/hydrocephalus). In 2015 FBCNS published the abstracts from ISHCSF annual meeting in Banff, Canada (http://www.fluidsbarrierscns.com/supplements/12/S1). We anticipate this new affiliation will lead to an increase in journal content in basic research into hydrocephalus and other CSF disorders and will stimulate further research leading to new ideas and treatment options for devastating neurological disorders. Our international editorial board has been expanded to reflect the affiliation with ISHCSF by strengthening the membership with expertise in neurosurgery, neuro- imaging and neurological disorders, including hydrocephalus.

## The future

There have been many recent advances in research on the brain barriers and fluids of the brain. Development of novel techniques, such as in imaging and better in vitro models should help in those advances. There is also a greater understanding of the importance of such research in disease states and treatments. FBCNS, with its focus on basic research into barriers, brain fluid dynamics and associated diseases, aims to foster such advances. The affiliations with IBBS and ISHCSF will help in achieving that aim.
